# Current perspectives on psychedelic treatments in Europe

**DOI:** 10.1016/j.lanepe.2025.101537

**Published:** 2025-11-20

**Authors:** Santiago Madero, Oscar Soto-Angona, Genis Ona, Jose Sanchez-Moreno, Eduard Vieta

**Affiliations:** a Department of Medicine, School of Medicine & Health Sciences, University of Barcelona (UB), 143 Casanova St., 08036, Barcelona, Catalonia, Spain; b Bipolar and Depressive Disorders Unit, Hospital Clinic, 170 Villarroel St., 08036, Barcelona, Catalonia, Spain; c Institut d’Investigacions Biomèdiques August Pi i Sunyer (IDIBAPS), 170 Villarroel St., 08036, Barcelona, Catalonia, Spain; d Institute of Neurosciences (UBNeuro), Barcelona, Spain; e Centro de Investigación Biomédica en Red de Salud Mental (CIBERSAM), Instituto de Salud Carlos III, Madrid, Spain; f ANIMA Group, Institut de Recerca Sant Joan de Déu (IRSJD), Santa Rosa 39-57, 08950, Esplugues de Llobregat, Spain; g Parc Sanitari Sant Joan de Déu, Dr. Antoni Pujades 42, 08830, Sant Boi de Llobregat, Spain; h Department of Psychiatry and Forensic Medicine, Universitat Autónoma de Barcelona, Cerdanyola del Vallès, Spain; i Sociedad Española de Medicina Psicodélica (SEMPsi), Barcelona, Spain; j Universitat Rovira i Virgili, Departament de Psicologia, 43007, Tarragona, Spain

**Keywords:** Psychedelic-assisted therapy, Regulatory developments, Methodological hallenges in psychedelic clinical trials

## Abstract

In this viewpoint, we explore the evolving landscape of psychedelic-assisted therapy in Europe, focusing on clinical, regulatory, and therapeutic developments. While access remains limited, recent initiatives in Switzerland, Germany, and the Czech Republic illustrate growing momentum toward regulated use. We examine the debate surrounding psychedelics as pharmacological agents versus psychotherapeutic catalysts and argue for an integrative framework that considers neurobiological mechanisms, subjective experience, and contextual factors. We focus on how their effects, particularly those involving neuroplasticity and critical periods, may interact with psychotherapeutic processes. We highlight the importance of context and psychological support in shaping outcomes and discuss the challenges of implementing scalable care models. Regulatory fragmentation and methodological complexities continue to hinder progress, but publicly funded trials such as EPIsoDE and PsyPal offer promising examples of ethical and effective approaches. In our view, the future of psychedelic therapy lies not in simplifying its complexity, but in embracing it.

## Introduction

Despite growing public interest and media attention, no major regulatory authority has approved psychedelic substances for clinical use in any specific indication. Access remains limited in most countries to clinical trials, off-label use, or special access programs.^1^ Nonetheless, psychedelics are increasingly perceived as viable therapeutic options, particularly among patients for whom conventional treatments are ineffective or unavailable, fueled by positive media coverage and a gradual reduction in stigma. At the same time, academic and industry-led research has expanded rapidly, supported by emerging regulatory pathways that begin to define formal requirements for psychedelic therapies.^2^

In this context, classical psychedelics such as psilocybin, N,N-dimethyltryptamine (DMT), and the atypical psychedelic 5-methoxy-DMT (5-MeO-DMT), or the empathogen/entactogen 3,4-methylenedioxymethamphetamine (MDMA), have re-emerged as potential therapeutic tools. Preliminary evidence suggests these compounds may allow for rapid and lasting improvements in conditions such as depression, post-traumatic stress disorder (PTSD), anxiety, and so-called “existential distress”.^3^, ^4^, ^5^ Their mechanisms appear to depend not only on pharmacological effects but also on the quality of the subjective experience, challenging conventional psychiatric models and drug regulation.

This viewpoint examines how different European countries are regulating and delivering psychedelic treatments, and explores the debate over their classification as pharmacological agents, psychotherapeutic tools, or both. We analyze comparative care models, emerging neurobiological insights, and the regulatory complexities shaping access and implementation. Rather than endorsing a single model, we advocate for an integrative perspective that considers the interplay between drug effects, psychological processes, and contextual factors, essential for developing safe, effective, and scalable psychedelic-assisted care frameworks in Europe.

## Status of psychedelics in Europe

Recent developments in Switzerland, Germany, and the Czech Republic illustrate the growing momentum toward regulated access.

Switzerland has implemented a limited medical use program since 2014, allowing authorized physicians to treat patients with MDMA, LSD, and psilocybin under strict regulatory oversight. By 2024, over 700 patients with depression, PTSD, and anxiety disorders have received treatment. The program includes preparatory, treatment, and integration sessions. While psychotherapy is not legally required, it's commonly integrated. The Swiss model is notable for its decentralized, physician-led structure and emphasis on professional societies and training.^6^

Germany recently approved its first Compassionate Use Program for psilocybin, a milestone in EU psychedelic regulation. Allowing adult patients with treatment-resistant depression access to psilocybin under exceptional circumstances, overseen by the Central Institute of Mental Health in Mannheim,^7^ reflecting growing institutional support for early access pathways.

In June 2025 the Czech Republic's lower house passed a landmark bill enabling medical psilocybin access under specific conditions, later ratified by the Senate, and signed into law. Treatment for depression, PTSD, and substance use disorders, must be administered by qualified psychiatrists in clinical settings with assisted psychotherapy.^8^ This positions the Czech Republic at the forefront of pragmatic, medically supervised psychedelic policy.

## Pharmacological tools or psychotherapeutic catalysts

Among current debates on psychedelic therapy, a key question is whether these substances should be prescribed as standalone agents with psychological support or viewed primarily as psychotherapy assisted by psychedelics. Each stance implies a preference for either neurobiological models or approaches emphasizing psychological and contextual factors. However, psychedelics may offer a bridge between these often opposing views.

Biomedically, psychedelics are considered to possess distinct neurobiological mechanisms that may exert therapeutic effects independent of psychotherapy. Evidence shows transdiagnostic efficacy across mental health conditions, even with varied therapeutic interventions. Historically, most interventions are non-directive supportive rather than structured psychotherapy, leading some to argue psychotherapy may not be essential.^9^ This aligns with regulatory models focused on efficacy and safety of a drug compared to placebo. Supporting this, a recent trial showed efficacy of 5-MeO-DMT in TRD without any accompanying psychotherapeutic intervention,^10^ though findings remain preliminary.

Conversely, intensity and quality of psilocybin-induced experiences seem to predict symptom improvement more reliably than dose.^11^ Research highlights mystical-type experiences^12^ and emotional breakthroughs^13^ as key predictors of outcomes. Evidence seems to point psychedelic experiences being context dependent, as supportive environments with appropriate psychological preparation increase the likelihood of positive experiences and uncontrolled or risky settings heighten the risk of challenging or distressing reactions.^14^ Psychological interventions could help shape these acute effects, which appear central to therapeutic benefit. Beyond acute phase, psychedelics may exert their effect through enhanced psychological flexibility,^15^ improved therapeutic alliance,^16^ and increased capacity for meaning making,^17^ all facilitated by psychotherapy.

Recent findings show two high-dose psilocybin sessions, with psychological support, outperform a six-week selective serotonin reuptake inhibitor (SSRI) course in reducing negative cognitive biases associated with depression.^18^ This underscores the importance of context and short-term, experience-driven interventions, prompting a reevaluation of how efficacy is measured in mental health. However, necessity of psychotherapy remains debated.

Regulatory standards are still evolving. Although the role of psychotherapy in enhancing treatment efficacy remains under debate,^9^^,^^19^ if the psychedelic experience proves to be an inherent aspect, psychological support or psychotherapy is essential for safety, posing implementation challenges. First, widespread implementation would require major infrastructure investment and specialized training for clinicians, raising concerns about cost-effectiveness and accessibility. Second, consensus on the optimal integration of psychotherapeutic interventions with psychedelic administration is lacking and research remains limited due to methodological challenges in comparing diverse interventions.

## Comparative models for delivery of care

The structure and implementation of psychotherapies within psychedelic treatments remain subjects of ongoing debate. Proposed models, range from evidence-based therapies such as cognitive-behavioral therapy (CBT) or acceptance and commitment therapy (ACT), to non-structured or non-manualized approaches often labeled as “psychological support” or “non-directive psychotherapy”.^20^

Despite differences, most models share three core phases: (a) preparation, in which non-drug sessions are conducted to build rapport and reduce anxiety; (b) drug administration, typically a day-long session in a controlled setting; and (c) integration, where the psychological material that emerged is processed and incorporated into the patient's life.^21^

A key distinction often overlooked is between structured psychotherapeutic modalities and psychological support. While some industry actors label intervention as psychological support to avoid regulatory hurdles, these interventions often rely on common factors of psychotherapy, such as empathy, therapeutic alliance, and reflective listening which require professional training. Cavarra et al. (2022) emphasize that common factors across therapeutic models are central to outcomes, especially in psychedelic therapy where suggestibility and emotional openness amplify their impact. The therapeutic stance, whether directive or non-directive, can influence the patient's experience and integration process.^22^ Knudsen (2025) notes that while many psychedelic trials include preparation and integration sessions, extent of psychological support vary. Some offer minimal support; others embed comprehensive psychotherapeutic frameworks. This variability has implications for both efficacy and accessibility. Importantly, Knudsen distinguishes between psychedelic-assisted psychotherapy, which involves structured therapeutic engagement, and psychologically assisted psychedelic therapy, where the drug's pharmacological effects are primary, and support is limited to safety and emotional containment.^23^

Therefore, labeling these interventions as mere “support” may obscure their therapeutic nature. Even non-directive approaches involve psychotherapeutic processes and should be treated as such in terms of training, oversight, and ethical standards. This distinction is especially relevant as psychedelic therapy moves toward broader clinical implementation, where clarity around therapeutic roles and competencies will be crucial. Moreover, the challenges in defining and manualizing psychotherapy are not unique to psychedelic trials. Common factors are inherently difficult to standardize and have long posed challenges across psychotherapy research. Psychedelic therapy inherits these complexities rather than introduces them uniquely, and any regulatory or scientific framework must account for this broader context.

In real-world settings, the integration of psychotherapy into psychedelic treatment varies across European models. In Switzerland's limited access program, psychotherapy is commonly included but not legally mandated, allowing for flexibility in clinical practice. Similarly, Germany's Compassionate Use Program reflects a flexible stance, prioritizing patient safety and clinical oversight without imposing rigid therapeutic frameworks.^7^ In contrast, the Czech Republic has taken a more standardized approach: regulatory agencies have mandated that psilocybin administration for therapeutic use must follow an officially recognized clinical recommended procedure for assisted psychotherapy.^8^

## Neurobiology meets psychology

Over the last years, the neuroplasticity-enhancing effect of psychedelics has played a central role when explaining their potential therapeutic use. These findings could reconcile debates about the role of psychotherapy and relevance of subjective effects in psychedelic therapies.

Several models explore how neuroplasticity changes interact with interventions. One approach focuses on the modulation of “critical periods”, understood as periods of heightened sensitivity to stimuli, allowing increased biological and behavioral malleability. Preliminary data suggest psychedelics may reopen these periods.^24^ Although more research is needed, this framework offers a promising way to understand neuroplasticity enhancement by psychedelics. It bridges biology and environment by showing how environmental responses manifest as neurobiological changes, with outcomes depending on context. In that regard, the undirected susceptibility model is especially relevant.^25^ According to this model, undirected approaches (e.g. psychedelic drugs), unlike classical psychopharmacology, would promote the susceptibility to change (re-opening critical periods) in a non-directed way. Which may require psychotherapeutic guidance to retrieve schemas for their subsequent modification.

A preclinical study showed MDMA reinstated critical periods only in social contexts, not in isolated animals,^26^ underscoring the importance of context. Thus, hypothetically, pharmacological intervention alone may not fully harness neuroplasticity's benefits without synergistic environments. In this view, even when if psychedelic drugs without subjective effect are developed, the neuroplasticity changes might still need psychotherapeutic interventions to allow this malleability to translate into positive outcomes. This needs to be proven and should be tested experimentally.

## Regulatory challenges, market access and opportunities in Europe

The complexity of psychedelic mechanisms mirrors the challenges faced in defining appropriate regulatory pathways. As of 2025, the clinical development of psychedelic therapies is advancing rapidly, with most late-stage trials driven by industry actors in the United States, in part because its pharmaceutical market is financially appealing and the FDA has issued formal guidance for psychedelic drug development, providing a centralized and predictable regulatory framework.^27^ Although Europe has a more complex and fragmented landscape, the new EU Health Technology Assessment Regulation (HTAR),^28^ in force since January 2025, aims to harmonize clinical assessments, decisions on pricing and reimbursement remain decentralized. Each Member State conducts separate evaluations, leading to duplication, inconsistent outcomes, and delays in access, particularly for innovative treatments like psychedelics.

The EMA's updated guidance on clinical investigation for depression now formally includes psychedelics, particularly for treatment-resistant depression (TRD), calling for rigorous, placebo-controlled trial designs and long-term safety monitoring.^29^ However, psychedelics pose unique regulatory challenges due to their subjective effects, sensitivity to context, methodological issues related to functional unblinding and expectancy bias. These issues extend into Health Technology Assessment (HTA) processes, which prioritize cost-effectiveness and real-world value. The case of esketamine (not formally a psychedelic) illustrates these challenges: although approved by the EMA in 2019, uptake has been limited due to HTA skepticism over benefits, high cost, and supervised administration requirements. For classic psychedelics, which may require psychological support and specialized infrastructure, these barriers are even greater and often overlooked in cost-utility models.^30^ Nonetheless, national initiatives are beginning to address these gaps. Germany's Compassionate Use Program, Switzerland's physician-led limited access framework, and the Czech Republic's recent legislation enabling psilocybin therapy are key steps toward establishing regulated psychedelic care in Europe. These models offer insights into responsibly implementing early access programs while generating real-world data.

Despite systemic obstacles, publicly funded European trials are pushing forward. The EPIsoDE study, supported by Germany's Federal Ministry of Education and Research and the MIND Foundation, is the country's first randomized, double-blind, placebo-controlled trial assessing psilocybin for TRD. It enrolled 144 patients and combined pharmacological sessions with structured psychotherapy.^31^ Likewise, EU Funded PsyPal project explores psilocybin-assisted therapy for existential distress in palliative care across nine European countries.^32^ It reflects growing recognition of psychedelics' role in end-of-life care and a shift toward more compassionate, holistic models of care. Both initiatives uphold rigorous research standards and ethical oversight while addressing often overlooked clinical questions, such as applications in terminal illness and mental health resilience.

## Conclusion

Psychedelic therapies are not just pharmacological interventions, they can be viewed as experiential catalysts that challenge conventional boundaries between drug and context, biology and meaning, regulation and care. Their therapeutic potential lies in the interplay between neurobiological effects and the psychological, relational, and environmental frameworks in which they are delivered. Attempts to isolate the drug from its setting, or remove the therapeutic scaffolding for regulatory simplicity, risk undermining the very mechanisms that could contribute to the effectiveness of these treatments.

A key unresolved issue is the durability of treatment effects. While some studies report sustained improvements after one or two sessions, long-term outcomes remain uncertain. Questions persist around retreatment frequency, relapse management, and how to proceed when initial sessions fail. Current clinical paradigms are underdeveloped, and optimal dosing, frequency, and retreatment strategies require systematic investigation.

Although more evidence is needed on the use of psychedelics without therapeutic interventions, there is broad consensus supporting the added value of psychotherapeutic support for safety and efficacy. Group-based models may improve accessibility and cost-effectiveness. Psychotherapy research could help identify shared mechanisms across therapeutic modalities and evaluate specific therapy–drug combinations. Preliminary studies have begun to explore this, but more research is needed to establish evidence-based guidelines for integrating psychotherapy, if necessary, with psychedelic treatments.^33^

Limited access programs in Switzerland, Germany, and the Czech Republic offer unique opportunities to address these gaps. These programs are not merely transitional, they offer clinicians, researchers, and regulators a window to observe how psychedelic therapies function in practice and collect real-world data on safety, efficacy, and implementation, informing the future regulatory frameworks that reflect the realities of clinical care.

In our viewpoint, the future of psychedelic therapy depends not on reducing complexity, but on learning to work with it. It will require a shift from reductionism to integration; from asking “what works?” to asking, “what works, for whom, and under what conditions?”. Only by embracing this complexity we can build a model of care that effective, ethical, inclusive, and grounded in the lived experience of healing.

## Contributors

All authors contributed equally to this manuscript.

## Declaration of interests

SM has received CME-related honoraria, or consulting fees from Open Health, BeckleyPsytech, Janssen-Cilag, Lundbeck, Lundbeck/Otsuka, and Viatris, with no financial or other relationship relevant to the subject of this article. EV has received grants and served as consultant, advisor or CME speaker for the following entities: AB-Biotics, Abbott, AbbVie, Adamed, Adium, Alcediag, Angelini, Biogen, Beckley-Psytech, Biohaven, Boehringer-Ingelheim, Casen-Recordati, Celon Pharma, Compass, Dainippon Sumitomo Pharma, Esteve, Ethypharm, Ferrer, Gedeon Richter, GH Research, Glaxo-Smith Kline, HMNC, Intra-Cellular therapies, Idorsia, Johnson & Johnson, Lundbeck, Luye Pharma, Menarini, Medincell, Merck, Mitsubishi Tanabe Pharma, Newron, Novartis, Organon, Orion Corporation, Otsuka, Roche, Rovi, Sage, Sanofi-Aventis, Sunovion, Takeda, Teva, and Viatris, outside the submitted work. OSA declares no conflicts of interest. GO declares no conflicts of interest. JS declares no conflicts of interest.

## References

[1] Butlen-Ducuing F., Silva F., Silva I., Balabanov P., Thirstrup S. (2025). Applying the EU regulatory framework for the clinical use of psychedelics. Lancet Psychiatry.

[2] Oliveira-Maia A.J., Seybert C. (2025). Dilemmas in psychedelic medicine: from ethics to regulation and equity. Eur Neuropsychopharmacol.

[3] Ghaznavi S., Richter S.G. (2025). Classic psychedelics for the treatment of depression: potential benefits and challenges. Drugs.

[4] Amaev A., Song J., Kambari Y. (2025). The effects of psychedelic-assisted therapy on illness and death anxiety: a systematic review and meta-analysis. J Psychiatr Res.

[5] Sze Jing Yong A., Bratuskins S., Sultani M.S., Blakeley B., Davey C.G., Bell J.S. (2025). Safety and efficacy of methylenedioxymethamphetamine (MDMA)-assisted psychotherapy in post-traumatic stress disorder: an overview of systematic reviews and meta-analyses. Aust N Z J Psychiatry.

[6] Liechti M.E., Gasser P., Aicher H.D., Mueller F., Hawrot T., Schmid Y. (2025). Implementing psychedelic-assisted therapy: history and characteristics of the Swiss limited medical use program. Neurosci Appl.

[7] Zentralinstitut für Seelische Gesundheit (2025). Compassionate use program for psilocybin possible for the first time in Germany: ZI Mannheim. https://www.zi-mannheim.de/en/institute/news/compassionate-use-program-for-psilocybin-possible-for-the-first-time-in-germany.html.

[8] Parliament of the Czech Republic (2025).

[9] Goodwin G.M., Malievskaia E., Fonzo G.A., Nemeroff C.B. (2024). Must psilocybin always “Assist Psychotherapy”?. Am J Psychiatry.

[10] (2025). GH research. https://investor.ghres.com/news-releases/news-release-details/gh-research-announces-primary-endpoint-met-phase-2b-trial-gh001.

[11] Goodwin G.M., Aaronson S.T., Alvarez O. (2025). The role of the psychedelic experience in psilocybin treatment for treatment-resistant depression. J Affect Disord.

[12] Ko K., Knight G., Rucker J.J., Cleare A.J. (2022). Psychedelics, mystical experience, and therapeutic efficacy: a systematic review. Front Psychiatry.

[13] Roseman L., Haijen E., Idialu-Ikato K., Kaelen M., Watts R., Carhart-Harris R. (2019). Emotional breakthrough and psychedelics: validation of the emotional breakthrough inventory. J Psychopharmacol.

[14] Carhart-Harris R.L., Roseman L., Haijen E. (2018). Psychedelics and the essential importance of context. J Psychopharmacol.

[15] Davis A.K., Barrett F.S., Griffiths R.R. (2020). Psychological flexibility mediates the relations between acute psychedelic effects and subjective decreases in depression and anxiety. J Contextual Behav Sci.

[16] Levin A.W., Lancelotta R., Sepeda N.D. (2024). The therapeutic alliance between study participants and intervention facilitators is associated with acute effects and clinical outcomes in a psilocybin-assisted therapy trial for major depressive disorder. PLoS One.

[17] Hartogsohn I. (2018). The meaning-enhancing properties of psychedelics and their mediator role in psychedelic therapy, spirituality, and creativity. Front Neurosci.

[18] Henry J., Giribaldi B., Nutt D.J., Erritzoe D., Carhart-Harris R., Lyons T. (2025). The effects of psilocybin therapy versus escitalopram on cognitive bias: a secondary analysis of a randomized controlled trial. Eur Neuropsychopharmacol.

[19] Gründer G., Brand M., Mertens L.J. (2024). Treatment with psychedelics is psychotherapy: beyond reductionism. Lancet Psychiatry.

[20] Ram A.V., Abraham K.L., Storch E.A. (2025). Evidence-based therapy models in a new age of psychedelic-assisted psychotherapy. J Cogn Psychother.

[21] Brennan W., Belser A.B. (2022). Models of psychedelic-assisted psychotherapy: a contemporary assessment and an introduction to EMBARK, a transdiagnostic, trans-drug model. Front Psychol.

[22] Cavarra M., Falzone A., Ramaekers J.G., Kuypers K.P.C., Mento C. (2022). Psychedelic-assisted psychotherapy—a systematic review of associated psychological interventions. Front Psychol.

[23] Knudsen G.M. (2025). Classical psychedelics in therapy: essential psychotherapy or minimal support?. Eur Neuropsychopharmacol.

[24] Nardou R., Sawyer E., Song Y.J. (2023). Psychedelics reopen the social reward learning critical period. Nature.

[25] Branchi I. (2011). The double edged sword of neural plasticity: increasing serotonin levels leads to both greater vulnerability to depression and improved capacity to recover. Psychoneuroendocrinology.

[26] Nardou R., Lewis E.M., Rothhaas R. (2019). Oxytocin-dependent reopening of a social reward learning critical period with MDMA. Nature.

[27] Food and Drug Administration (FDA) (2023). Psychedelic drugs: considerations for clinical investigations guidance for industry draft guidance. https://www.fda.gov/drugs/guidance-compliance-regulatory-information/guidances-drugs.

[28] Official Journal of the European Union (2021).

[29] European Medicines Agency (2025). Committee for Medicinal Products for Human Use (CHMP) guideline on clinical investigation of medicinal products in the treatment of depression. http://www.ema.europa.eu/contact.

[30] Butlen-Ducuing F., McCulloch D.E.W., Haberkamp M. (2023). The therapeutic potential of psychedelics: the European regulatory perspective. Lancet.

[31] Mertens L.J., Koslowski M., Betzler F. (2022). Methodological challenges in psychedelic drug trials: efficacy and safety of psilocybin in treatment-resistant major depression (EPIsoDE) – rationale and study design. Neurosci Appl.

[32] PsyPal (2024). Clinical trial into psilocybin therapy for psychological distress in palliative care, PsyPal, receives medical ethical authorisation, with conditions. https://palliativeprojects.eu/psypal/2024/12/17/psypal-press-release-17-12-24/.

[33] Beaussant Y., Tarbi E., Nigam K. (2024). Acceptability of psilocybin-assisted group therapy in patients with cancer and major depressive disorder: qualitative analysis. Cancer.

